# Pathological Complete Response Achieved with Colorectal Cancer-Based Chemotherapy for Locally Recurrent Cecal Neuroendocrine Carcinoma after Surgery: A Case Report

**DOI:** 10.70352/scrj.cr.25-0633

**Published:** 2026-02-04

**Authors:** Kana Kajisako, Taro Tanabe, Naho Nishibayashi, Sachiko Ishida, Suguru Ogihara, Takahiro Hobo, Koji Kobayashi, Hirokazu Toshima, Ken Shimada, Noboru Yokoyama, Haruhiro Inoue

**Affiliations:** 1 Digestive Diseases Center, Showa Medical University Koto Toyosu Hospital, Tokyo, Japan; 2 Department of Oncology, Showa Medical University Koto Toyosu Hospital, Tokyo, Japan

**Keywords:** neuroendocrine carcinoma, colon cancer, pathological complete response, CEA, chemotherapy

## Abstract

**INTRODUCTION:**

Primary neuroendocrine carcinoma (NEC) of the colon is extremely rare, accounting for only 0.2% of all colorectal malignancies, and is associated with a poor prognosis. Early diagnosis is often challenging, as endoscopic biopsies are frequently misinterpreted as adenocarcinoma. Although platinum-based regimens such as etoposide plus cisplatin or irinotecan are commonly used, no standard chemotherapy protocol has been established. We report a case of locally recurrent cecal NEC that responded remarkably to a colorectal cancer–based chemotherapy regimen, achieving a pathological complete response.

**CASE PRESENTATION:**

A 52-year-old man presented with severe anemia. Imaging and colonoscopy revealed a cecal tumor initially diagnosed as adenocarcinoma. He underwent laparoscopic right hemicolectomy with lymph node dissection. Final pathology revealed NEC, staged as pT3N2aM0, Stage IIIB. Adjuvant etoposide–cisplatin chemotherapy was initiated. Three months postoperatively, carcinoembryonic antigen (CEA) rose to 44.4 ng/mL, and CT demonstrated a perianastomotic peritoneal nodule and lymphadenopathy, consistent with recurrence. Considering the adenocarcinoma component in the primary tumor and elevated CEA, chemotherapy was switched to a colorectal cancer–based regimen: FOLFOXIRI plus bevacizumab. After 7 cycles, both radiologic regression and normalization of CEA levels were achieved. Resection of the recurrent lesions confirmed a pathological complete response with no residual tumor cells.

**CONCLUSIONS:**

To the best of our knowledge, this is the first reported case of cecal NEC achieving pathological complete regression with a colorectal cancer–based chemotherapy regimen. Our findings indicate that colorectal NEC may respond not only to platinum-based regimens but also to colorectal cancer–based regimens. Furthermore, CEA levels may serve as a clinically relevant biomarker to guide chemotherapy selection in this setting.

## Abbreviations

CEA
: carcinoembryonic antigen
EP
: etoposide and cisplatin
MANEC
: mixed adeno-neuroendocrine carcinoma
NEC
: neuroendocrine carcinoma

## INTRODUCTION

Primary neuroendocrine carcinoma (NEC) of the colon is a rare entity, representing approximately 0.2% of all colorectal malignancies, and is associated with an extremely poor prognosis.^1)^ Early and accurate diagnosis remains challenging, as endoscopic biopsy specimens are frequently misinterpreted as adenocarcinoma.^2–4)^ While no standardized treatment protocol has been established, multidisciplinary approaches combining surgical resection and adjuvant chemotherapy are commonly employed. Platinum-based regimens with etoposide or irinotecan are generally used as first-line chemotherapy, yet a standard regimen has not been established.^5,6)^ Herein, we report a case of locally recurrent cecal NEC that was successfully treated with a colorectal cancer–based chemotherapy regimen, resulting in a pathological complete response.

## CASE PRESENTATION

**Fig. 1:**
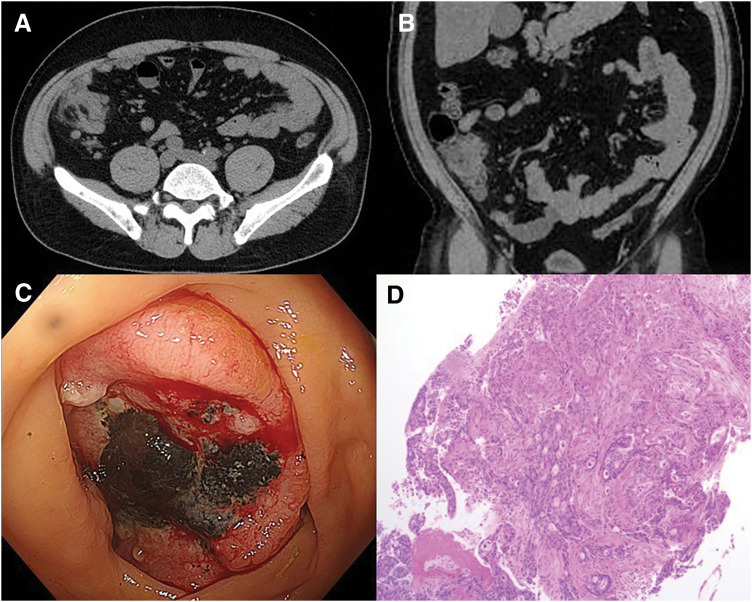
Imaging and histological findings. (**A**, **B**) Abdominal CT demonstrated irregular wall thickening of the cecum accompanied by regional lymphadenopathy, with no evidence of distant metastasis. (**C**) Lower gastrointestinal endoscopy revealed a type 2, semicircumferential lesion on the side opposite the ileocecal valve. (**D**) Histopathological diagnosis revealed moderately differentiated adenocarcinoma (tub2) predominant over well-differentiated adenocarcinoma (tub1).

A 52-year-old man with no significant past medical history was emergently admitted with lightheadedness. Laboratory investigations on admission revealed severe anemia with a hemoglobin level of 6.0 g/dL. Abdominal CT demonstrated irregular wall thickening of the cecum along with increased density of the surrounding adipose tissue (**Fig. 1A** and **1B**). Colonoscopy revealed a tumor in the cecum (**Fig. 1C**), and biopsy results led to a diagnosis of advanced cecal adenocarcinoma (**Fig. 1D**). Genetic analysis revealed that both RAS and BRAF were wild-type, and microsatellite instability was negative. The patient subsequently underwent laparoscopic right hemicolectomy with lymph-node dissection.

**Fig. 2:**
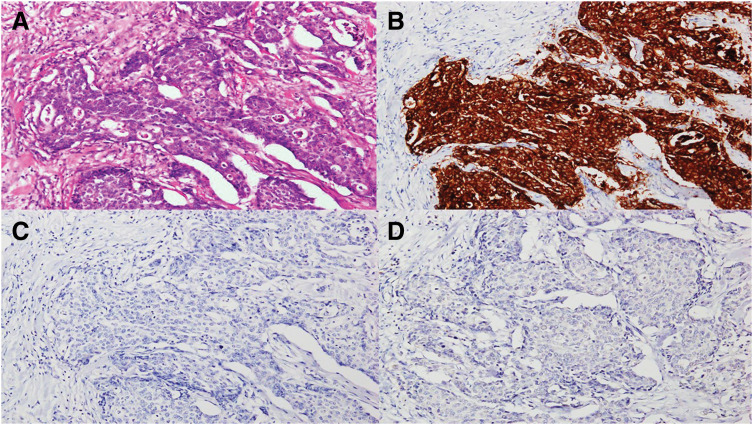
Histological findings of the tumor. (**A**) H&E staining revealed tumor cells with a higher N/C ratio compared to conventional adenocarcinoma. (**B**) Immunohistochemical staining for synaptophysin was diffusely positive. (**C**, **D**) Both chromogranin A and CD56 were negative on immunohistochemical staining. H&E, Hematoxylin and eosin; N/C, nuclear-to-cytoplasmic

**Fig. 3:**
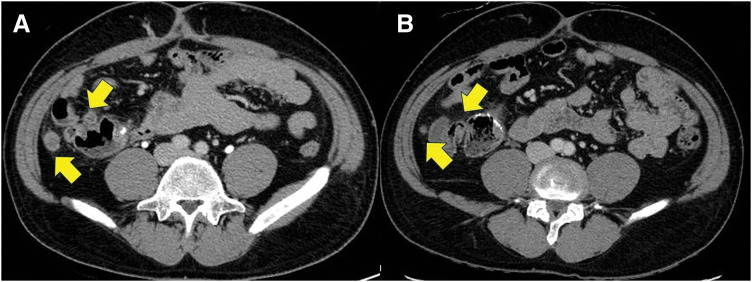
Imaging findings before and after chemotherapy. Yellow arrows shows a disseminated nodule and a swelling lymph node. (**A**) Follow-up CT after initiation of EP therapy revealed peritoneal dissemination nodules adjacent to the anastomosis site and enlargement of the surrounding lymph nodes. (**B**) CT after 6 courses of Bmab/FOLFOXIRI revealed a marked reduction in both the peritoneal dissemination nodules and lymph node enlargement. Bmab, bevacizumab; EP, etoposide and cisplatin

Histopathological examination of the surgical specimen revealed a diagnosis of NEC, staged as pT3N2aM0 (Stage IIIB, according to UICC TNM classification, 8th edition). Although an adenocarcinoma component was identified, it accounted for less than 30% and did not meet the criteria for mixed adeno-neuroendocrine carcinoma (MANEC) (**Fig. 2A**–**2D**). Postoperatively, the patient received 1 course of adjuvant chemotherapy with the etoposide and cisplatin (EP) regimen. However, 3 months after surgery, serum carcinoembryonic antigen (CEA) levels rose to 44.4 ng/mL, whereas the preoperative CEA was 12.3 ng/mL. Subsequent CT revealed a perianastomotic peritoneal nodule and lymphadenopathy, suggestive of local recurrence (**Fig. 3A**).

**Fig. 4:**
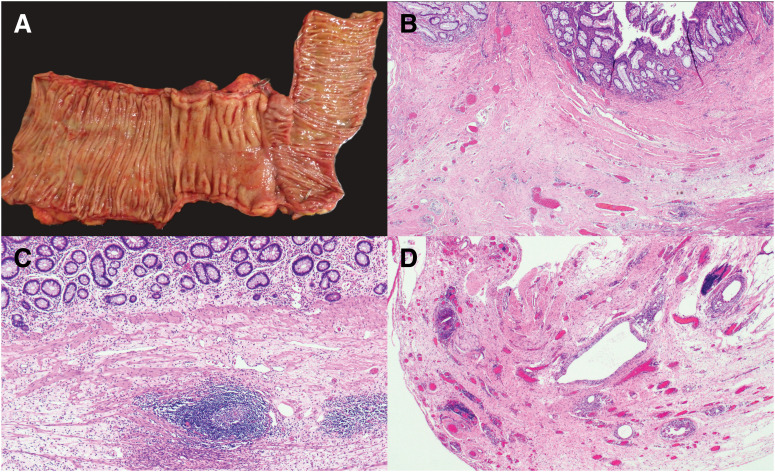
Macroscopic and histopathological findings of the resected specimen after surgery for anastomotic recurrence. (**A**) Gross appearance of the resected specimen. No obvious mass-forming lesion is identified. (**B**) The submucosal layer shows extensive fibrosis. No residual tumor is observed, findings consistent with treatment-related changes. (**C**) Edema and fibrosis are present in the submucosal layer, with scattered lymphoid follicles. (**D**) Numerous foreign-body–type multinucleated giant cells and foamy macrophages are observed. These findings are compatible with reactive changes induced by treatment.

Given the adenocarcinoma component in the primary specimen and the elevated CEA level, chemotherapy was switched from EP to a colorectal cancer–based regimen consisting of bevacizumab plus FOLFOXIRI. After 7 cycles, marked regression of both the peritoneal nodule and lymphadenopathy was observed (**Fig. 3B**), along with normalization of CEA levels. There was no evidence of other distant metastasis. Curative resection of the recurrent lesions was performed (**Fig. 4A**), and histopathological analysis confirmed a pathological complete response with no residual tumor cells (grade 3 therapeutic effect) (**Fig. 4B**–**4D**). At 6 months postoperatively, the patient remains recurrence-free.

## DISCUSSION

We encountered a case of locally recurrent NEC of the colon, detected during adjuvant chemotherapy, in which a colon cancer–based chemotherapy regimen achieved a pathological complete response. This case provides 2 key insights. First, colorectal NEC may respond not only to the generally recommended platinum-based chemotherapy regimens^7–9)^ but also to colon cancer–based chemotherapy in selected cases. Second, CEA elevation may reflect adenocarcinoma-like biological behavior in a subset of colorectal NEC, which could potentially aid in therapeutic decision-making.^10)^

According to the National Comprehensive Cancer Network guidelines, EP regimens, based on protocols for small cell lung carcinoma, are recommended for NEC.^11)^ However, bevacizumab combined with FOLFOX6, based on treatment protocols for colorectal carcinoma, has also been applied in clinical practice, and several reports, in addition to the present case, have documented favorable outcomes with colon cancer–based regimens in colorectal NEC.^12,13)^

The histogenesis of colorectal NEC has been proposed to involve 4 possible origins: (i) pre-existing adenocarcinoma, (ii) pre-existing carcinoid tumor, (iii) non-neoplastic multipotent stem cells, and (iv) non-neoplastic immature neuroendocrine cells. Among these, adenocarcinoma-derived NEC is reported to be the most common.^14)^ This provides a rationale for the potential effectiveness of colon cancer–based chemotherapy in NEC of adenocarcinoma origin.

Furthermore, colorectal NEC has been reported to exhibit elevated expression of vascular endothelial growth factor (VEGF) compared with conventional advanced colorectal carcinoma.^15)^ Preclinical studies have demonstrated that VEGF inhibition can suppress tumor growth and angiogenesis in NEC models, suggesting a possible therapeutic role for anti-VEGF agents such as bevacizumab. However, clinical evidence supporting this strategy remains limited, and its efficacy should be interpreted with caution.

In the present case, although the adenocarcinoma component was <30% and thus insufficient for a diagnosis of MANEC, the elevated CEA level during recurrence strongly suggested an adenocarcinoma origin. This may explain the profound therapeutic effect of colon cancer–based chemotherapy despite the minimal histological adenocarcinoma component.

## CONCLUSIONS

Although platinum-based regimens following protocols for small cell lung carcinoma are currently recommended for colorectal NEC, our case demonstrated marked efficacy of colon cancer–based chemotherapy. These findings suggest that consideration of the biological characteristics and possible cellular origin of colorectal NEC may be important for treatment selection. Serum CEA levels may provide supportive information reflecting adenocarcinoma-like features in a subset of cases; however, their role as a biomarker remains exploratory. Further accumulation of clinical data is required to establish optimal treatment strategies for colorectal NEC.

## DECLARATIONS

### Availability of data and materials

All data on which the conclusions of this case report are based are included in the present publication.

## References

[1] Moertel CG. Karnofsky memorial lecture. An odyssey in the land of small tumors. J Clin Oncol 1987; 5: 1502–22. 2443618 10.1200/JCO.1987.5.10.1502

[2] Shafqat H, Ali S, Salhab M, et al. Survival of patients with neuroendocrine carcinoma of the colon and rectum: a population-based analysis. Dis Colon Rectum 2015; 58: 294–303. 25664707 10.1097/DCR.0000000000000298

[3] Silva DJ, Dos Santos J, Vaz AP, et al. Rectal mixed adenoneuroendocrine carcinoma: Case report. Medicine (Baltimore) 2021; 100: e27348. 34622834 10.1097/MD.0000000000027348 PMC8500653

[4] Watanabe J, Suwa Y, Ota M, et al. Clinicopathological and prognostic evaluations of mixed adenoneuroendocrine carcinoma of the colon and rectum: a case-matched study. Dis Colon Rectum 2016; 59: 1160–7. 27824701 10.1097/DCR.0000000000000702

[5] Morizane C, Machida N, Honma Y, et al. Effectiveness of etoposide and cisplatin vs irinotecan and cisplatin therapy for patients with advanced neuroendocrine carcinoma of the digestive system: the TOPIC-NEC Phase 3 randomized clinical trial. JAMA Oncol 2022; 8: 1447–55. 35980649 10.1001/jamaoncol.2022.3395 PMC9389440

[6] Zhang P, Li J, Li J, et al. Etoposide and cisplatin versus irinotecan and cisplatin as the first-line therapy for patients with advanced, poorly differentiated gastroenteropancreatic neuroendocrine carcinoma: A randomized phase 2 study. Cancer 2020; 126(Suppl 9): 2086–92. 32293725 10.1002/cncr.32750 PMC7186825

[7] Bernick PE, Klimstra DS, Shia J, et al. Neuroendocrine carcinomas of the colon and rectum. Dis Colon Rectum 2004; 47: 163–9. 15043285 10.1007/s10350-003-0038-1

[8] Conte B, George B, Overman M, et al. High-grade neuroendocrine colorectal carcinomas: a retrospective study of 100 patients. Clin Colorectal Cancer 2016; 15: e1–7. 26810202 10.1016/j.clcc.2015.12.007 PMC4885752

[9] Smith JD, Reidy DL, Goodman KA, et al. A retrospective review of 126 high-grade neuroendocrine carcinomas of the colon and rectum. Ann Surg Oncol 2014; 21: 2956–62. 24763982 10.1245/s10434-014-3725-3 PMC4521622

[10] Owaki S, Mori Y, Nakai S, et al. BRAF V600E-mutated colorectal neuroendocrine carcinoma effectively treated with a chemotherapy protocol for BRAF-mutated metastatic colorectal cancer. Intern Med 2024; 63: 1995–9. 37981300 10.2169/internalmedicine.2870-23 PMC11309870

[11] Shah MH, Goldner WS, Benson AB, et al. Neuroendocrine and Adrenal Tumors, Version 2.2021, NCCN Clinical Practice Guidelines in Oncology. J Natl Compr Canc Netw 2021; 19: 839–68. 34340212 10.6004/jnccn.2021.0032

[12] Garcia-Carbonero R, Sorbye H, Baudin E, et al. ENETS Consensus Guidelines for High-Grade Gastroenteropancreatic Neuroendocrine Tumors and Neuroendocrine Carcinomas. Neuroendocrinology 2016; 103: 186–94. 26731334 10.1159/000443172

[13] Hadoux J, Malka D, Planchard D, et al. Post-first-line FOLFOX chemotherapy for grade 3 neuroendocrine carcinoma. Endocr Relat Cancer 2015; 22: 289–98. 25770151 10.1530/ERC-15-0075

[14] Ogimi T, Sadahiro S, Kamei Y, et al. Distribution of neuroendocrine marker-positive cells in colorectal cancer tissue and normal mucosal tissue: consideration of histogenesis of neuroendocrine cancer. Oncology 2019; 97: 294–300. 31390635 10.1159/000501521 PMC6888884

[15] Rodríguez-Remírez M, Del Puerto-Nevado L, Fernández Aceñero MJ, et al. Strong antitumor activity of bevacizumab and aflibercept in neuroendocrine carcinomas: in-depth preclinical study. Neuroendocrinology 2020; 110: 50–62. 31030198 10.1159/000500591

